# Pureed diets containing a gelling agent to reduce the risk of aspiration in elderly patients with moderate to severe dysphagia: A randomized, crossover trial

**DOI:** 10.1097/MD.0000000000021165

**Published:** 2020-07-31

**Authors:** Reiko Kyodo, Takahiro Kudo, Akira Horiuchi, Torao Sakamoto, Toshiaki Shimizu

**Affiliations:** a Department of Pediatrics, Juntendo University Faculty of Medicine, Tokyo; b Digestive Disease Center; c Department of Rehabilitation, Showa Inan General Hospital, Komagane, Japan.

**Keywords:** aspiration pneumonia, deglutition, dysphagia, gelling agent, pureed diet

## Abstract

**Goals:**

The aim of this study was to evaluate the effectiveness of adding a gelling agent to pureed diets to prevent aspiration pneumonia in elderly patients with moderate to severe dysphagia.

**Background:**

Pureed diets are often used to reduce aspiration in patients with dysphagia. However, the ideal texture of a pureed diet to prevent aspiration pneumonia remains unclear.

**Materials and methods:**

We prospectively conducted a randomized, crossover trial of pureed rice with or without a gelling agent in patients with moderate to severe dysphagia (ClincialTrials.gov number, NCT03163355). The primary outcome measure was pharyngeal residuals using an endoscopic scoring system. The secondary outcome was the sense of material remaining in the throat following swallowing.

**Results:**

Sixty two patients (58% men), mean age 83 ± 9 years with moderate to severe dysphagia were included. Residuals in the throat were significantly less likely with pureed rice with than without the gelling agent (median cyclic ingestion score (range); 1 (0-4) vs 2 (0-4); *P* = .001). Irrespective of the presence or absence of the gelling agent, the sense of material remaining in the throat was significantly less frequent in older patients (87 ± 7.6 vs 75 ± 9.1 years, *P* < .01; 86 ± 7.3 vs 75 ± 8.6 years, *P* < .01).

**Conclusions:**

Pureed diets containing a gelling agent may reduce the risk of aspiration pneumonia possibly by decreasing pharyngeal residues in elderly patients with moderate to severe dysphagia.

## Abstract 2

Supplemental Digital Content is available in the text

## 1 Introduction

Dysphagia is estimated to affect 8% of the world population (590 million people). Texture-modified foods and thickened drinks are commonly used to reduce the risks of choking and aspiration.^[1]^ The International Dysphagia Diet Standardisation Initiative (IDDSI) was founded with the goal of developing globally standardized terminology and definitions for texture-modified foods and liquids applicable to individuals with dysphagia of all ages, in all care settings, and all cultures.^[1]^ One approach has been to use texture-modified dysphagia diets to improve swallowing ability and provide nutrition tor patients with moderate to severe dysphagia susceptible to aspiration.^[2]^ Previous studies with pureed diets have shown that dysphagia patients can often meet their nutritional requirements and avoid enteral feeding via percutaneous endoscopic gastrostomy tube.^[3]^

Thickening agents are frequently used in patients with dysphagia.^[2,3]^ A thickening agent or thickener is a substance which increases the viscosity of a liquid without substantially changing its other properties. The goal of thicken liquids is to slow the flow of liquids to allow more time for airway closure. However, very thick liquids and solid foods may also require greater effort in terms of the propulsive forces of the tongue needed to drive the bolus through the oropharynx^[4]^ and thus potentially increase the risk of residual remaining in the recesses of the pharynx after swallowing. The presence of pharyngeal residue is thought to be an important factor for the development of aspiration pneumonia.^[5,6]^

Some thickening agents are also gelling agents which when dissolved in the liquid phase as a colloid mixture form a weakly cohesive internal structure. Recently, starch degrading enzymes have been added to gelling agents with the goal of reducing adherence of pureed diets to the pharynx.^[7]^ We have successfully used gelling agents with the texture of pureed rice in our hospital since 2016 based on the hypothesis that the texture of the pureed diet is likely to a factor predictive of the development of aspiration pneumonia. However, the ideal texture of a pureed diet to prevent aspiration pneumonia remains unclear.^[8]^ The aim of this study was to evaluate the effectiveness of pureed diets containing a gelling agent for the prevention of aspiration pneumonia in elderly patients with moderate to severe dysphagia.

## 2 Materials and methods

### 2.1 Study design

All studies were done at the Showa Inan General Hospital, which is a local municipal hospital in Japan. The Institutional Review Board of Showa Inan general Hospital approved the study protocol on January 27, 2017. All subjects or their guardians gave written informed consent for this study which was a prospective randomized cross-over trial comparing pharyngeal residues of pureed diet with and without a gelling agent in elderly patients with dysphagia. The study was reported according to the CONSORT guidelines and was registered at www.clinicaltrials.gov (NCT03163355) on May 1, 2017. Randomization was performed using software (Epidat 4.2, 2016) which generated random codes assigned to participants.

### 2.2 Subjects

From May 2017 to September 2018 hospitalized patients who underwent endoscopic swallowing evaluation were consecutively enrolled. Subjects were included irrespective of whether oral intake of dysphagic diets was considered successful or unsuccessful. Exclusion criteria included age less than 65 years old or the presence of an acute infection. Patient who had developed new cerebrovascular disease, myocardial infarction, aspiration pneumonia within 2 weeks were also excluded.

### 2.3 Study protocol

Gastroenterologists experienced in transnasal esophagogastroduodenoscopy performed endoscopic swallowing evaluation along with a speech therapist. The degree of dysphagia was evaluated using the Hyodo-Komagane score which ranges from 0 to12 and were scored as mild (0–3), moderate (4–7), or severe (8–12).^[9]^ Subsequently, a randomized, crossover trial using pureed rice containing the gelling agent, or the same pureed rice diet without the gelling agent was performed in patients with dysphagia. The extent of pharyngeal residue after swallowing of pureed rice with or without jelly was evaluated using the cyclic ingestion score (Table 1). Both the investigators and subjects did not know which diet was used.

**Table 1:**
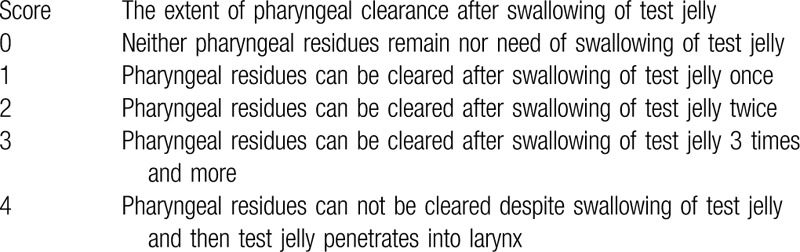
Endoscopic cyclic ingestion score.

In addition, the sense of material remaining in the throat (scored as present or absence) after swallowing of pureed rice with or without the test jelly was asked of the patients using yes/no question during the procedure.

### 2.4 Procedures

Participant underwent endoscopic swallowing evaluation while sitting in a chair or sitting up in bed. Two minutes prior to inserting the endoscope, 0.2 to 0.5 ml of 4% lidocaine was applied to the nasal cavities of each participant as a nasal spray. An endoscope (GIF-XP260N, Olympus, Tokyo, Japan) was used for endoscopic swallowing evaluation. This is a forward viewing upper gastrointestinal video scope with an ultra-miniature, resolution charged-coupled device with a 120-degree field of view. The insertion diameter is 5.5-mm and the video scope has a tip deflection capability of 210/120 up/down in a single plane. The lubricated endoscope was passed transnasally, typically on the floor of nose, to obtain a superior view of the hypopharynx. The endoscope was moved between swallowing and the post swallow position to collect the data as described previously.^[9,10]^ Images of the oropharynx and larynx were displayed on a monitor and recorded on the digital video recorder (Olympus, HVO-3300MT).

### 2.5 Test diets

The pureed rice containing the gelling agent was prepared by mixing 100 g of rice porridge at >70°C with 0.5 g of the gelling agent (Softia U, Nutri Co., Ltd., Yokkaichi, Japan) with a blender for a minute. The texture characteristics (IDDSI Level 4) were: hardness, 1760 ± 125 N/m^2^; cohesiveness, 0.59 ± 0.03; adhesiveness, 224 ± 56 J/m^3^. The pureed rice without the gelling agent was made by mixing 100 g of white rice and 50 ml of water with a blender for a minute. The texture characteristics (IDDSI Level 4) were: hardness, 312 ± 11.3 N/m^2^; cohesiveness, 0.81 ± 0.02; adhesiveness, 108 ± 5.8 J/m^3^. For patients with pooling in the vallecula and piriform sinuses after swallowing pureed diets, cyclic ingestion with 3 ml of jelly consisting of gelatin jelly (Isotonic green jelly, Nutri Co., Ltd., Yokkaichi, Japan) was performed to assist with pharyngeal clearance. The characteristics were as follows: Hardness, 5000 N/m^2^; cohesiveness, 0.4; adhesiveness, 89 J/m^3^.

### 2.6 Evaluations

The primary outcome measure was the presence of material remaining in the throat scored using the endoscopic cyclic ingestion score (0−4). The secondary outcome measure was the sense of material remaining in the throat (scored as present or absent) after the swallowing of pureed rice and/or test jelly. Supplemental Digital Content can be seen (Video 1 shows an example of the pureed rice with gelling agent. Pharyngeal residues were cleared after swallowing of test jelly once (score 1)), (Video 2 shows the pureed rice without gelling agent pooled in the vallecula and piriform sinuses. Pharyngeal residues can be cleared after swallowing of test jelly twice (score 2)).

### 2.7 Statistical analysis

Each value shows mean ± SD or median (range). For the study we estimated (from preliminary data) that cyclic ingestion score in the standard pureed diet group without gelling agent would be 2 or 3. For a 50% improvement in the pureed diet group containing gelling agent, a total of at least 58 subjects in each arm would be required (alpha 0.05, beta 0.2). Categorical data were compared by the χ^2^-test or Fisher exact test where appropriate. Numerical data were analyzed by the Student *t* test. Non-parametric data were analyzed by Wilcoxon rank sum test. In addition, logistic regression analysis was applied to identify independent predictors of a sense of material remaining in the throat after the swallowing of pureed rice and/or test jelly. Statistical significance was taken as a two-sided *P* value <.05. Statistical analysis was performed by using JMP 9.0.2 version software (SAS Institute Inc.).

## 3 Results

A total of 70 patients were enrolled; 62 patients (58% men), mean age 83 ± 9 years with dysphagia were included in the final analysis. Eight patients were excluded due to new cerebrovascular disease, myocardial infarction, aspiration pneumonia within 2 weeks. Their demographic and clinical date are shown in Table 2. Severe comorbid diseases, such as aspiration pneumonia (35%), cerebrovascular disease (31%), others (34%) were present. Moderate to severe dysphagia was confirmed as 87% of the enrolled patients had a Hyodo-Komagane score ≥4.

**Table 2:**
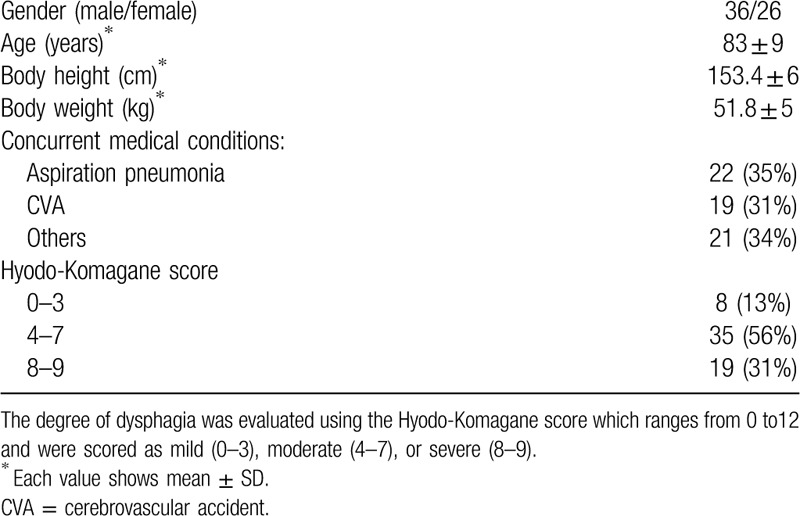
Clinical features of 62 patients with dysphagia who were enrolled in this study.

**Figure 1:**
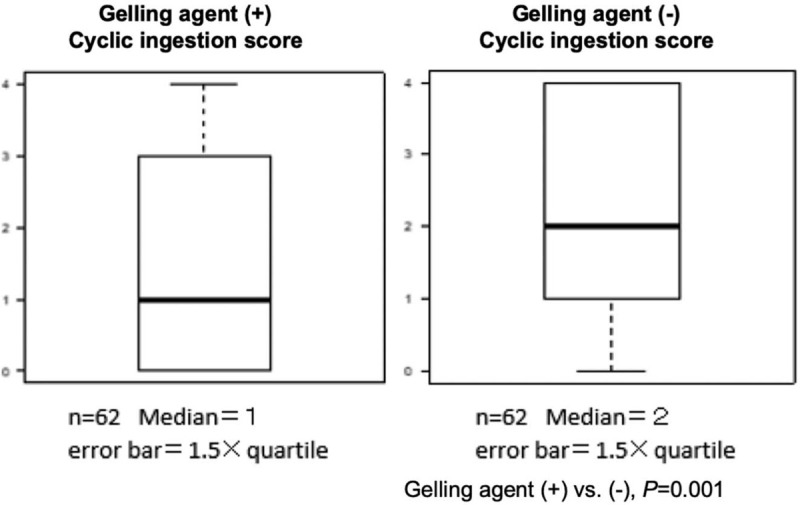
Comparison of median cyclic ingestion scores after swallowing pureed diet with and without gelling agent using endoscopic examination of swallowing.

Pureed rice with the gelling agent had a median cyclic ingestion score (range) significantly lower than when given without the gelling agent (1 (0–4) vs 2 (0–4), *P* = .001) (Fig. 1). A greater proportion, 42% (26/62) were able to swallow pureed rice with a gelling agent without pooling in the vallecula and piriform sinuses (cyclic ingestion score = 0) compared to the standard pureed rice without a gelling agent without pooling in the vallecula and piriform sinuses (42% vs 16%, *P* = .002) (Table 3). On the other hand, patients with a pharyngeal clearance score of 2 (the score is obtained after swallowing of test jelly twice) was significantly less than those receiving standard pureed rice without a gelling agent (8% vs 21%, *P* = .041) (Table 3).

**Table 3:**
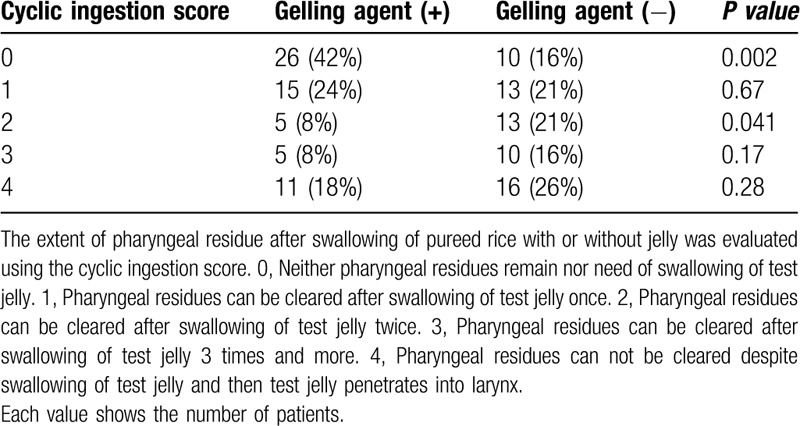
Comparison of cyclic ingestion score after swallowing of pureed diet with or without gelling agent.

In patients in whom the pharyngeal residues were present there was no significant difference in the sense of material remaining in the throat, independent of whether the gelling agent was administered (71% vs 77%, *P* = .41) (Table 4). Irrespective of the presence or absence of the gelling agent, the sense of material remaining in the throat was significantly less frequent in older patients (87 ± 7.6 vs 75 ± 9.1 years, *P* < .01; 86 ± 7.3 vs 75 ± 8.6 years, *P* < .01). By logistic regression analysis the sense material of remaining in the throat after swallowing of pureed rice was significantly related to the age of patients, irrespective of the use of gelling agent (Table 5).

**Table 4:**
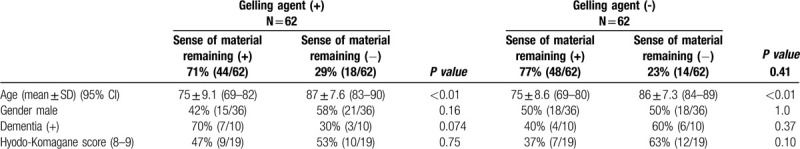
The characteristics of a sense of material remaining in the throat after swallowing of pureed diet with or without gelling agent.

**Table 5:**
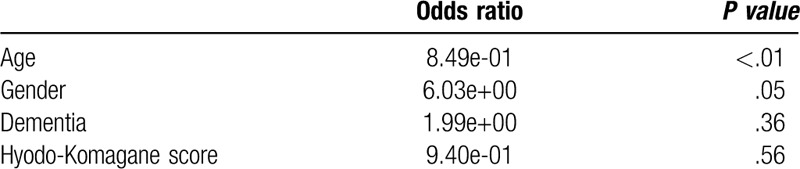
Multiple regression analysis of a sense of material remaining in the throat after swallowing of pureed diet with or without gelling agent.

### 3.1 Adverse events

No adverse events such as cardiopulmonary evens or aspiration pneumonia occurred in subjects of the prospective study.

## 4 Discussion

Pureed diets are commonly used as an initial dysphagia diets for patients with moderate to severe dysphagia. The use of a pureed diet containing a gelling agent was associated with decreased pharyngeal residues and was associated with a reduction in aspiration pneumonia in the elderly with moderate to severe dysphagia. The sense of material remaining in the throat after swallowing of the pureed diet was significantly less frequent in older patients, irrespective of the texture of the pureed diet (Table 5). The lack of sensing material remaining in the throat may help explain why the very elderly more readily develop aspiration pneumonia. Our data suggests that puree diets containing a gelling agent may help prevent aspiration in the elderly with moderate to severe dysphagia possibly by reducing pharyngeal residues.

The advantage of pureed diets containing a gelling agent has been thought to be related to the decrease in adhesiveness compared to standard pureed diets without a gelling agent. However, in this study the adhesiveness of pureed rice containing the gelling agent was actually greater than that of pureed rice without the gelling agent (224 ± 56 vs 108 ± 5.8 J/m^3^). It is possible that in vitro differences in adhesiveness may less reflect its tendency to stick to the mucosa.

Altering the characteristics of food is one of the most common approaches used to help compensate for dysphagia.^[11]^ Size, color, shape, taste, viscosity, and texture are all components used to characterize food. In general, thin fluids, such as water, soup, or juice are more likely to be aspirated^[12]^; therefore, thickening fluids using thickeners is 1 mainstay of dysphagia diets.^[13]^ One study of 66 dysphagic patients documented that thickened fluid and a soft mechanical diet reduced the incidence of pneumonia by 80% compared with a regular diet.^[14]^ Despite these advantages, many patients and caregivers are reluctant to prescribe a dysphagia diet, especially a pureed diet. For example, in 1 study, 75% of the patients who were recommended to modify the pureed diet with thickeners did not prefer using them.^[15]^ The pureed rice we used that contained a gelling agent that required mixing porridge at >70°C and thus the preparation of pureed diet containing gelling agent may also require more effort compared with the preparation of a standard pureed diet.

This study has some limitations. Although this study was not blinded, both the investigators and subjects did not know which diet was used for testing. In addition to a relatively small sample size, the study was conducted in a single hospital and will need to be confirmed in multicenter studies, with different populations and possibly different pureed diets as the only diet tested with or without gelling agent was rice as it is the staple diet in Japan. Other kinds of pureed food with gelling agent should be compared with those without gelling agent to clarify the advantage of addiction of gelling agent to pureed food. In addition, the inter- and intra-rater reliability of the Hyodo-Komagane score and endoscopic cyclic ingestion score was not measured.

In conclusion, purred diets containing a gelling agent appeared to reduce the risk of aspiration pneumonia possibly by decreasing pharyngeal residues in elderly patients with moderate to severe dysphagia.

## Acknowledgments

The authors thank Prof. David Y. Graham for his editorial assistance and assistance with English.

## Author contributions

R.K. and A.H. designed the study and wrote the manuscript; R.K., T.K, A.H. and T.S. collected and analyzed the data. A.H. and T.S. revised the manuscript critically for important intellectual content. All authors read and approved the final manuscript.

## Supplementary Material

Supplemental Digital Content

## Supplementary Material

Supplemental Digital Content
